# *Rorippa indica* Regeneration via Somatic Embryogenesis Involving Frog Egg-like Bodies Efficiently Induced by the Synergy of Salt and Drought Stresses

**DOI:** 10.1038/srep19811

**Published:** 2016-01-22

**Authors:** Kedong Xu, Yunxia Chang, Yi Zhang, Kun Liu, Ju Zhang, Wei Wang, Zhanshuai Li, Jianxin Wu, Shuya Ma, Yuexing Xin, Chunjing Li, Qianbei Zhou, Hanhan Qiu, Yumei Pi, Youwei Wang, Guangxuan Tan, Chengwei Li

**Affiliations:** 1 Key Laboratory of Plant Genetics and Molecular Breeding, Zhoukou Normal University, Zhoukou, 466001, People’s Republic of China; 2 College of Life Science and Agronomy, Zhoukou Normal University, Zhoukou, 466001, People’s Republic of China

## Abstract

Frog egg-like bodies (FELBs), novel somatic embryogenesis (SE) structures first observed in *Solanum nigrum*, were induced in *Rorippa indica*. NaCl-mediated salt and mannitol-mimicked drought stresses induced FELBs in *R*. *indica*, which is very different from the induction by plant growth regulators (PGRs) under low light condition that was used in *S*. *nigrum* FELB induction. It demonstrated that NaCl or mannitol supplements alone could induce FELBs in *R*. *indica*, but with low induction rates, while the synergy of NaCl and mannitol significantly increased the FELB induction rates. For the combination of 5.0 g/L mannitol and 10.0 g/L NaCl the highest FELB induction rate (100%) was achieved. It suggests that the synergy of drought and salt stresses can replace PGRs to induce FELBs in *R*. *indica*. On medium supplemented with 1.0 mg/L gibberellic acid all the inoculated *in vitro* FELBs developed into multiple plantlets. Morphological and histological analyses confirmed the identity of FELBs induced in *R*. *indica* and revealed that FELBs originate from root cortex cells.

*Rorippa indica* is an annual cruciferous herb distributed worldwide. In China it is used as a kind of wild vegetable and traditional medicine in China^1^. *R*. *indica* is a rich resource of valuable medicinal components, among others of glucosinolates, flavonoids, roripamine and isothiocyanates^1^,^2^,^3^. Mustard aphid, *Lipaphis erysimi*, threatens the cultivation of the rapeseed-mustard (*Brassica* spp.) crop, and till present in cultivated *Brassica* germplasm no resistance was found. As a result, no resources are available for mustard aphid resistance breeding of *Brassica* crops. *R*. *indica* was found to be resistant to the aphid^4^, and therefore it could serve as a resistance resource.

*R*. *indica*, used as medicinal and vegetable in China, can be used as a model for research in cruciferous plants next to *Arabidopsis thaliana*, for its small adult stature, short life cycle, small genome size, prolific seed production and self-pollination^5^,^6^. In addition, the growth conditions for *R*. *indica* are less demanding than those for *A. thaliana*.

However, as *R*. *indica* has not much studied as a plant species, high-frequency regeneration and transformation systems in *R*. *indica* have not been established. Frog egg-like bodies (FELBs), which were first observed in *Solanum nigrum*^7^, are novel structures of somatic embryogenesis (SE). Regeneration via SE involving FELBs is highly efficient and labor saving, which makes it suitable for germplasm preservation and establishment of transformation systems^7^. Generally plant growth regulators (PGRs), like 2,4-dichlorophenoxyacetic acid (2,4-D) or 1-naphthaleneacetic acid (NAA), are necessary for the induction of SE. In the presence of PGRs, desiccation, drought, and salt stresses could promote regeneration efficiency^8^,^9^,^10^,^11^. However, the effects of these stresses on plant regeneration in the absence of PGRs have not been reported. In this study, an *R*. *indica* regeneration system via SE involving FELBs was first established via a new method. In this system the synergy of salt and drought stresses in the absence of PGRs resulted in a high FELB induction rate (100%) in *R*. *indica*, and further led to high-efficiency SE-mediated plant regeneration. To our knowledge it is the first report that high-efficiency plant regeneration achieved by the synergistic effects of salt and drought stresses in the absence of PGRs.

## Results

### Under low light condition PGRs (NAA and 2,4-D) induce FELBs in *R*. *indica* with low FELB frequency

In the dark, none of the different combinations of concentrations of NAA and 2,4-D induces FELBs in *R. indica* root explants (Table 1). Therefore, a low light condition was employed instead. Under low light condition less than 1% of the *R*. *indica* root explants on media with the tested concentrations of NAA formed FELBs, while 2,4-D concentrations of 0.5, 1.0, and 1.5 mg/L resulted in FELB induction rates less than 19% (Table 1). This indicated that in low light condition FELBs can be induced at low rates in *R. indica* root explants.

### *R*. *indica* regeneration via SE involving FELBs on medium supplemented with mannitol and NaCl

**Figure 1:**
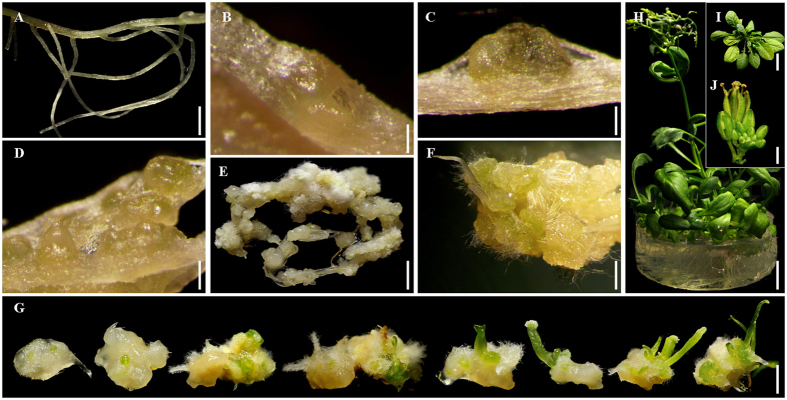
*R*. *indica* regeneration via SE involving FELBs. (**A–F**) The developmental process of FELBs induced from a root explant. (**A**) Root explant, scale bar = 0.5 cm. (**B**) FELBs formed under the epidermis of a root explant at an early developmental stage, scale bar = 200 μm. (**C**) Formed FELBs at an early-middle developmental stage, scale bar = 200 μm. (**D**) Formed FELBs at a middle stage of development, scale bar = 200 μm. (**E**) Formed FELBs at a late developmental stage, showing that nearly the entire root explant developed into FELBs, scale bar = 0.5 cm. (**F**) Enlarged view of FELBs from part of E, scale bar = 0.2 cm. (**G**) The process of separated *in vitro* FELB developing into multiple plantlets, scale bar = 1 cm. (**H**) Regenerated adult plants and seedlings, scale bar = 1 cm. (**I**) The rosette stage of a regenerated plant, scale bar = 1 cm. (**J**) The inflorescence of a regenerated plant, scale bar = 2 mm.

Two weeks after the induction of root explants (Fig. 1A), FELBs started to form under the epidermis of root explants, finally the epidermis burst by the development of FELBs (Fig. 1B–D). The induced FELBs in *R*. *indica* root explants were surrounded with a kind of special translucent sticky callus, which resembles the typical FELBs in *S*. *nigrum*^7^. At late developmental stages, clusters of mature FELBs containing multiple individual somatic embryoids surrounded with translucent sticky callus were formed (Fig. 1E,F). In most cases, one FELB gave rise to 5–10 individual somatic embryoids. It showed that 100.00% of the *in vitro* FELBs induced into multiple plantlets on MS medium supplemented with 1.0 mg/L GA_3_ within two weeks (Fig. 1G). The development of into multiple plantlets from *R*. *indica* root explants is similar in *S*. *nigrum*, but different from the regular patterns of traditional SE types, which one individual SE structure often develops into one plantlet^12^,^13^.

### Identification and morphological analysis of FELBs induced in *R*. *indica* root explants

**Figure 2:**
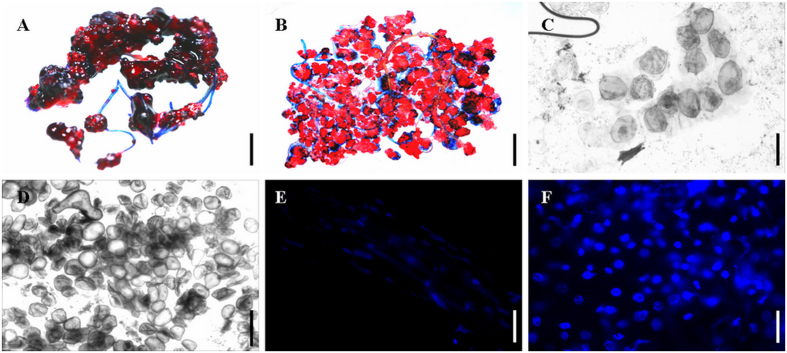
Identification and morphological analysis of FELBs induced in *R*. *indica*. (**A**) FELBs stained with acetocarmine and Evans blue, scale bar = 0.5 cm. (**B**) Microscopic squash specimen of FELBs stained with acetocarmine and Evans blue, scale bar = 0.5 cm. (**C**) Non-embryogenic callus part of FELBs stained with borax-toluidine blue, scale bar = 500 μm. (**D**) Embryogenic part of FELBs stained with borax-toluidine blue, scale bar = 500 μm. (**E**) Cell nuclei of the non-embryogenic callus cells in FELBs stained with DAPI and observed under dark-field lighting, scale bar = 500 μm. (**F**) Cell nuclei of the embryogenic cells in FELBs stained with DAPI and observed under dark-field lighting, scale bar = 500 μm.

Microscopic squash slides double stained with acetocarmine and Evans blue^7^,^14^ were employed to distinguish embryogenic from non-embryogenic callus part of FELBs, as embryogenic cells will be stained in red, and callus cells in blue. The images showed that the induced FELBs in *R*. *indica* root explants were composed of ball-shaped embryoids stained in red surrounded with translucent sticky callus stained in dark blue (Fig. 2A,B), similar to the FELBs induced in *S*. *nigrum*^7^. By using microscopic squash specimens together with borax-toluidine blue staining (Fig. 2C,D), and DAPI staining (Fig. 2E,F), the arrangement, size and cytoplasm thickness of FELB cells could be observed. This indicated that embryogenic cells of FELBs are smaller, with thicker cytoplasm, and more closely arranged compared to non-embryogenic callus cells of FELBs (Fig. 2B,E), which further inllustrated the embryogenic characteristics of the FELBs induced in *R*. *indica*.

### Histological detection of FELB origin and development

**Figure 3:**
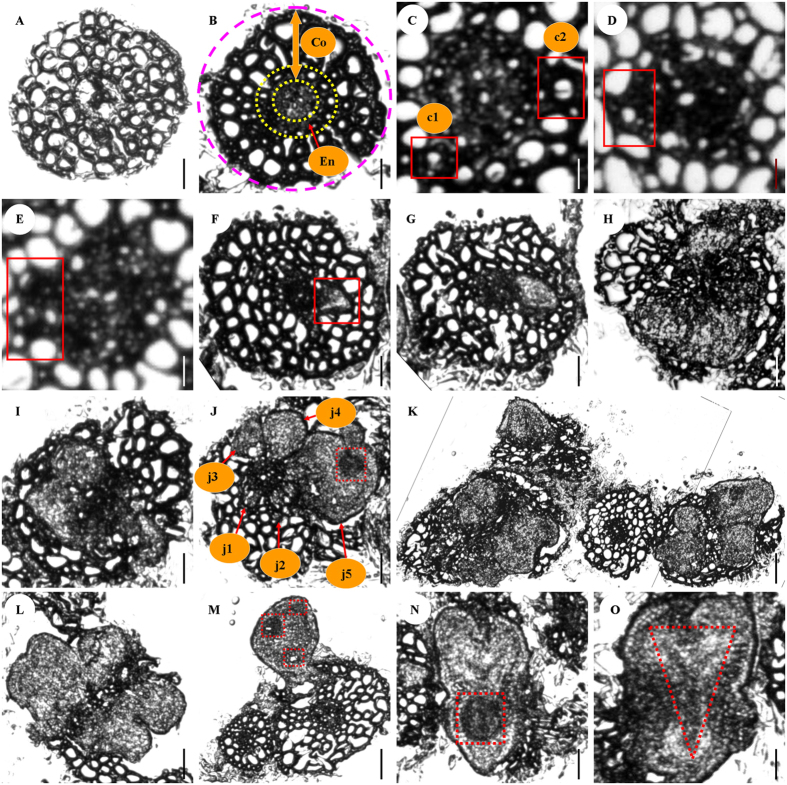
Microscopic frozen section images of *R*. *indica* FELBs at different developmental stages. (**A**) Transverse section of a root explant. (**B**) Transverse section of a root explant one day after inoculation, showing cortex (Co) between pink and inner yellow circles, and endodermis (En) between two yellow circles. (**C**) Single cells with thickened cell walls (marked with red frames c1 and c2) of endodermis in a root explant with the potential to develop into proembryos of FELBs. (**D,E**) Fast-division cells in formed proembryo rudiments (marked with red frames) of FELBs. (**F–M**) Formed FELBs containing proembryos and globular embryos at different developmental stages; (**J**) FELBs at different developmental stages (**j1–j5**) were induced in the same root explants, and the potential fast-cell-division zone (FCDZ) was formed in the j5 FELB (marked with a red frame); (**K**) FELBs formed in several neighbor root explants; (**M**) A FELB with three FCDZs (marked with red frames) burst the epidermis of the root explant. (**N**) Heart/torpedo-shaped embryo with a FCDZ (marked with a red frame) from a broken FELB burst by the embryo. (**O**) Cotyledon-shaped embryo with the rudiment of vascular tissue marked by a red triangle frames. Scale bars for (**A,B,F–J,L,N,O**), 200 μm. Scale bars for (**C–E**), 45 μm. Scale bar for (**K**) = 500 μm. Scale bar for (**M**) = 400 μm.

Frozen section technique was used to analyze the origin and development of FELBs. This clearly demonstrated that FELBs are derived from the root cortex (most FELBs are derived from root endodermis, the innermost layer of root cortex) (Fig. 3A–J), and that multiple embryoids are formed in individual FELBs (Fig. 3K–M). The stages, ranging from proembryo, globular, heart/torpedo-shaped embryos to cotyledon-shaped embryos were observed in FELBs at different developmental stages (Fig. 3F–M). Vascular tissues developed in FELB embryoids at late developmental stage (generally from cotyledon-shaped embryos on), while no vascular bundles connecting FELBs with parental tissues were formed (Fig. 3N,O). This observation suggests that the FELB vascular tissues were separated from the parental vascular tissues, further confirming the nature of *R*. *indica* FELB embryoids.

### Effects of mannitol-mimicked drought and NaCl-mediated salt stress on the induction of FELBs in *R*. *indica*

In the absence of PGRs, different combinations of a concentration series of 0, 2.5, 5.0, and 7.5 g/L of mannitol, and a concentration series of 0, 5.0, 10.0, and 15.0 g/L of NaCl, were tested to evaluate their effects on the induction of FELBs. FELB induction rates were very low (<1%) when only NaCl was supplemented to the media. FELB induction rates were lower than 19% if only mannitol was supplemented to the media (Table 2). However, the combination of mannitol and NaCl significantly increased the FELB induction rates (Table 2). The combination of 5.0 g/L mannitol and 10.0 g/L NaCl resulted in FELB induction rates till 100% of the inoculated root explants, highly significantly higher than all other tested concentration combinations of mannitol and NaCl (Table 2). The analysis of interaction effect between NaCl and mannitol indicated that there is interaction between NaCl and mannitol, and the combination of 5.0 g/L mannitol and 10.0 g/L NaCl also demonstrate highly significantly higher FELB induction rates than all other test concentration combinations (Table 3). These results suggest that mannitol and NaCl have a synergistic effect on FELB induction in *R*. *indica* root explants.

## Discussion

Supplementation of the optimal combination of PGRs in culture media is crucial to successful SE induction and plant regeneration^15^,^16^. In our previous study, FELBs, which are novel SE structures, were first induced in *S*. *nigrum*. 2,4-D and culturing of the explants in the dark were important factors for FELB induction in *S*. *nigrum*, while NAA was not suitable for FELB induction in *S*. *nigrum*^7^. In this study FELBs were also successfully induced by 2,4-D in *R*. *indica* root explants, similar to *S*. *nigrum*, while NAA was also found not suitable for FELB induction. Therefore, it seems that the effect of 2,4-D on FELB induction in contrast to that of NAA is more general. However, with same 2,4-D concentration gave rise to a much lower FELB induction rate in *R*. *indica* root explants compared to that of *S*. *nigrum*, suggesting that optimal concentrations of 2,4-D for FELB induction differ between plant species. *S*. *nigrum* and *R*. *indica* belong to Solanaceae and Cruciferae, respectively, illustrating that FELBs can be induced in plants from different families, highlighting the potential of FELB induction in other plant species. In this study, we also found that a low light condition other than the dark condition used in *S*. *nigrum* FELB induction^7^ was suitable for *R*. *indica* root explant FELB induction. It suggested that suitable light conditions for FELB induction are different among plant species.

Since the tested concentrations of 2,4-D did not result in high FELB induction rates, a new method was employed. For the new method, in the absence of PGRs, mannitol-mimicked drought stress and NaCl-mediated salt stress were adopted and successfully induced FELBs in *R*. *indica* root explants, moreover the synergy of NaCl and mannitol resulted in highly significant higher FELB induction rates than either of them alone (P < 0.01). The synergistic effect of mannitol and NaCl on somatic embryogenesis induction in *R*. *indica* was also much better than that of mannitol alone in *A. thaliana*^17^, and that of NaCl alone in *Vigna sinensis*^18^. The FELB induction rate induced by the synergistic optimal concentration combination of mannitol and NaCl reached 100.00%. These results show that drought and salt stresses can induce FELBs in *R*. *indica* root explants instead of PGRs and have synergistic effect on *R*. *indica* FELB induction. The mechanism of this synergistic induction is not clear.

In addition, in media supplemented with mannitol and NaCl, less contamination by microbes was observed during FELB induction (not published). A possible explanation is that supplements of NaCl and mannitol decreased the growth of potential contaminating microbes. This advantage will benefit the large-scale induction of FELB using this method, especially for the large-scale suspension cultures of FELB cells as bioreactor^7^.

The unavailability of a suitable transformation system for *R*. *indica* hampers its application as a model plant. The establishment of FELB mediated regeneration system will benefit the establishment of a transformation system for *R*. *indica*, which will facilitate model-plant applications and molecular breeding of *R*. *indica*.

## Materials and Methods

### Plant materials and explant preparation

*R*. *indica* seeds were treated with 75% (v/v) ethanol and 2.5% (v/v) sodium hypochlorite for sterilization, according to our published protocol^7^. For germination, the sterilized seeds were sown on 1/2 Murashige and Skoog (MS) medium^19^ supplemented with 1.0 mg/L gibberellic acid (GA_3_), 30 g/L sucrose, and 7.8 g/L agar (pH 5.8)^7^, then incubated in a germination chamber (25 °C in the dark) until the seeds were fully germinated. Seedlings were transplanted onto MS medium for the preparation of explants and cultured at 25 °C with a photoperiod of 16 h light (180 μmol·m^−2^s^−1^) and 8 h dark.

### Induction of FELBs

Following the protocol of FELB induction in *S*. *nigrum*^7^ NAA and 2,4-D with the concentration series 0, 0.5, 1.0, and 1.5 mg/L were employed to induce FELB in *R*. *indica*. The effect of mannitol-mimicked drought and NaCl mediated salt stress on *R*. *indica* root explant regeneration via FELBs mediated SE was tested. Root explants were placed on MS media with 30 g/L sucrose and 3.6 g/L gellan gum, pH 5.8, supplemented with different concentration combinations of mannitol (0, 2.5, 5.0, and 7.5 g/L) and NaCl (0, 5.0, 10.0, and 15.0 g/L). The explants were incubated at 25 °C and under a low light condition (36 μmol·m^−2^s^−1^) to induce FELBs. Different developmental stages of SE were recorded by using a digital camera (EOS 600D, Canon Inc., Japan) and a stereomicroscope (SMZ800, Nikon Corporation, Japan). To evaluate FELB induction rates, three hundred of root explants inoculated in thirty petri dishes (10 root explants inoculated in each petri dish) were calculated for each treatment.

### Histological and histochemical analyses of FELBs

Double staining with acetocarmine and Evans blue^20^ was employed to distinguish embryogenic cells from non-embryogenic ones. Embryogenic cells of FELBs were stained bright red, and non-embryogenic cells of callus and explants were stained dark blue^7^. Different SE stages were recorded by using a digital camera (EOS 600D, Canon Inc., Japan), a digital fluorescence microscope (BX 61, Olympus Corporation, Japan), and a digital optical microscope (BX 41, Olympus Corporation, Japan), respectively.

Staining with 4’,6-diamidino-2-phenylindole (DAPI) was used to detect the nuclei of embryonic and callus cells, following a previously published method^21^. Cell outlines were detected by using borax-toluidine blue staining^21^. Microscopic images were recorded by using a digital optical microscope (BX 41, Olympus Corporation, Japan). The frozen sections (thickness 8 μm) of FELBs at different developmental stages were made with a cryostat microtome (CM1850, Leica Microsystems, Germany) following a previously published method^5^. The sections were observed and recorded by using a digital optical microscope (BX 41, Olympus Corporation, Japan).

### Plantlet formation from *in vitro* FELBs

*In vitro* FELBs were placed on MS medium (pH 5.8) supplemented with 1.0 mg/L GA_3_, and cultivated at 25 °C under a photoperiod of 16 h light (180 μmol·m^−2^s^−1^) and 8 h dark. The formed plantlets with a length of 1-2 cm were separated and transferred onto 1/2 MS medium supplemented with 0.2 mg/L NAA, 30 g/L sucrose and 7.8 g/L agar (pH5.8) for root induction.

### Statistical analysis

The analysis of variance (ANOVA) was conducted on FELB induction rates of root explants in *R. indica* with 99% confidence intervals by using SPSS 16.0.

## Additional Information

**How to cite this article**: Xu, K. *et al*. *Rorippa indica* Regeneration via Somatic Embryogenesis Involving Frog Egg-like Bodies Efficiently Induced by the Synergy of Salt and Drought Stresses. *Sci. Rep*. **6**, 19811; doi: 10.1038/srep19811 (2016).

## Figures and Tables

**Table 1:** Effects of NAA and 2,4-D on FELB induction from root explants of *R*. *indica*.

NAA (mg/L)	2,4-D (mg/L)	Rate (%) of FELB induction	
		Dark condition	Low light
0		0.00 ± 0.00c	0.00 ± 0.00c
0.5		0.00 ± 0.00c	0.37 ± 0.12c
1.0		0.00 ± 0.00c	0.30 ± 0.12c
1.5		0.00 ± 0.00c	0.33 ± 0.12c
	0	0.00 ± 0.00c	0.00 ± 0.00c
	0.5	0.00 ± 0.00c	4.77 ± 0.62b
	1.0	0.00 ± 0.00c	18.70 ± 0.79a
	1.5	0.00 ± 0.00c	18.07 ± 0.83a

Note: FELB induction rate refers to the ratio of explants with induced FELBs to the total number of inoculated explants. The mean and standard error per treatment were calculated based on 300 explants from 30 petri dishes as 30 replicates (10 explants inoculated in each petri dish). Lowercase letters indicate highly significant differences at the 1% probability level. Highly significant differences were analyzed with one-way ANOVA by Tukey test using SPSS 16.0.

**Table 2:** Effects of mannitol and NaCl on FELB induction from root explants of *R*. *indica*.

NaCl (g/L)	Mannitol (g/L)	Rate (%) of FELB induction
0	0	0.00 ± 0.00k
	2.5	8.43 ± 0.23j
	5.0	18.73 ± 0.30i
	7.5	6.97 ± 0.29j
5.0	0	0.67 ± 0.05k
	2.5	47.10 ± 0.25e
	5.0	64.90 ± 0.42d
	7.5	38.30 ± 0.80f
10.0	0	0.57 ± 0.16k
	2.5	86.60 ± 0.62b
	5.0	100.00 ± 0.00a
	7.5	75.97 ± 0.41c
15.0	0	0.17 ± 0.09k
	2.5	25.40 ± 0.49h
	5.0	34.83 ± 0.45g
	7.5	8.13 ± 0.53j

Note: FELB induction rate refers to the ratio of explants with induced FELBs to the total number of inoculated explants. The mean and standard error per treatment were calculated based on 300 explants from 30 petri dishes as 30 replicates (10 root explants inoculated in each petri dish). Lowercase letters indicate highly significant differences at the 1% probability level. Highly significant differences were analyzed with one-way ANOVA by Tukey test using SPSS 16.0.

**Table 3:** Interaction effects between NaCl and mannitol on FELB induction from root explants of *R*. *indica*.

Induction factors	Concentration (g/L)	Rate (%) of FELB induction
NaCl	0	8.53d
	5.0	37.59b
	10.0	65.78a
	15.0	17.13c
Mannitol	0	0.20d
	2.5	41.88b
	5.0	54.62a
	7.5	32.34c

Note: FELB induction rate refers to the ratio of explants with induced FELBs to the total number of inoculated explants. The mean and standard error per concentration of NaCl or mannitol were calculated based on 4×300 explants from 4×30 petri dishes as 4×30 replicates (10 root explants inoculated in each petri dish), which is the total number of explants treated by the referred concentration of one of the stress elements combined with four different concentrations of the other stress element (There are 300 explants per combination from 30 petri dishes). Lowercase letters indicate highly significant differences at the 1% probability level. Highly significant differences were analyzed with two-way ANOVA by Tukey test using SPSS 16.0.
